# Prediction of acute coronary syndromes by urinary proteome analysis

**DOI:** 10.1371/journal.pone.0172036

**Published:** 2017-03-08

**Authors:** Nay M. Htun, Dianna J. Magliano, Zhen-Yu Zhang, Jasmine Lyons, Thibault Petit, Esther Nkuipou-Kenfack, Adela Ramirez-Torres, Constantin von zur Muhlen, David Maahs, Joost P. Schanstra, Claudia Pontillo, Martin Pejchinovski, Janet K. Snell-Bergeon, Christian Delles, Harald Mischak, Jan A. Staessen, Jonathan E. Shaw, Thomas Koeck, Karlheinz Peter

**Affiliations:** 1 Atherothrombosis and Vascular Biology, Baker IDI Heart and Diabetes Institute, Melbourne, Australia; 2 Department of Medicine, Monash University, Melbourne, Australia; 3 Clinical Diabetes and Epidemiology, Baker IDI Heart and Diabetes Institute, Melbourne, Australia; 4 Studies Coordinating Centre, Research Unit Hypertension and Cardiovascular Epidemiology, KU Leuven Department of Cardiovascular Sciences, University of Leuven, Leuven, Belgium; 5 Mosaiques Diagnostics GmbH, Hanover, Germany; 6 Sanford Burnham Prebys Medical Discovery Institute, La Jolla, California, United States of America; 7 Department of Cardiology, University Heart Centre Freiburg, Germany; 8 Department of Paediatrics, Stanford School of Medicine, Stanford, California, United States of America; 9 Barbara Davis Centre for Diabetes, University of Colorado School of Medicine, Aurora, Colorado, United States of America; 10 Institut National de la Santé et de la Recherche Médicale (INSERM), U1048, Institut of Cardiovascular and Metabolic Disease, Toulouse, France; 11 Université Toulouse III Paul-Sabatier, Toulouse, France; 12 Institute of Cardiovascular and Medical Sciences, University of Glasgow, Glasgow, United Kingdom; 13 R&D VitaK Group, Maastricht University, Maastricht, Netherlands; University Medical Center, GERMANY

## Abstract

Identification of individuals who are at risk of suffering from acute coronary syndromes (ACS) may allow to introduce preventative measures. We aimed to identify ACS-related urinary peptides, that combined as a pattern can be used as prognostic biomarker. Proteomic data of 252 individuals enrolled in four prospective studies from Australia, Europe and North America were analyzed. 126 of these had suffered from ACS within a period of up to 5 years post urine sampling (cases). Proteomic analysis of 84 cases and 84 matched controls resulted in the discovery of 75 ACS-related urinary peptides. Combining these to a peptide pattern, we established a prognostic biomarker named Acute Coronary Syndrome Predictor 75 (ACSP75). ACSP75 demonstrated reasonable prognostic discrimination (c-statistic = 0.664), which was similar to Framingham risk scoring (c-statistics = 0.644) in a validation cohort of 42 cases and 42 controls. However, generating by a composite algorithm named Acute Coronary Syndrome Composite Predictor (ACSCP), combining the biomarker pattern ACSP75 with the previously established urinary proteomic biomarker CAD238 characterizing coronary artery disease as the underlying aetiology, and age as a risk factor, further improved discrimination (c-statistic = 0.751) resulting in an added prognostic value over Framingham risk scoring expressed by an integrated discrimination improvement of 0.273 ± 0.048 (*P* < 0.0001) and net reclassification improvement of 0.405 ± 0.113 (*P* = 0.0007). In conclusion, we demonstrate that urinary peptide biomarkers have the potential to predict future ACS events in asymptomatic patients. Further large scale studies are warranted to determine the role of urinary biomarkers in clinical practice.

## Introduction

Coronary artery disease (CAD) and its complications such as acute coronary syndrome (ACS) are a leading cause of morbidity and mortality worldwide [1]. Several directions have been taken to search for the ideal methods for predicting future cardiovascular events, including simple clinical risk scoring systems such as the Framingham Risk Score and non-invasive techniques such as carotid intima-media thickness measurements by ultrasound. Novel alternative approaches have also been investigated, for example, fundus photography to measure retinal vessel calibers with narrower retinal arterioles and wider retinal venules conferring a greater risk of death, stroke and coronary artery disease in women [2]. The biomarker approach is one of the rapidly expanding areas in this field, starting from more traditional high density lipoprotein (HDL) cholesterol to high-sensitivity C-reactive protein (hsCRP), N-terminal pro-brain natriuretic peptide P (NT-proBNP), or even microparticles and microRNAs [3,4]. Reliable biomarkers to predict future ACS-events could lead to improved risk stratification, enable earlier interventions and potentially reduce the incidence of ACS. Current attempts include single biomarkers as well as biomarker combinations directed towards prediction of ACS specially or to CAD in general [5,6]. Yin et al. used mass spectrometry based plasma proteomics to identify protein biomarkers for the new onset of acute myocardial infarction (AMI) during a 3-year follow up in the Framingham Heart Study offspring cohort [5]. A multi-marker model composed of seven plasma proteins thereby reached a median C-statistic of 0.84 and exceeded models with regular clinical covariates.

Although this approach represents a first step towards predicting ACS, particularly AMI, based on proteomic biomarkers, routine blood proteomics can be challenging. This is due to the influence of processing, handling and storage of the specimen, and the potential instabilities of the proteome. We therefore used urine proteome analysis (UPA) for the identification of prognostic biomarkers for ACS. Proteome analysis of urine has been shown to be a rich and stable source of specific pathology-related information for multiple conditions including cardiovascular and renal diseases, revealing diagnostic and, more importantly, prognostic relevance [7–12]. It has been shown that UPA identified patients with coronary artery disease (CAD) using a diagnostic disease biomarker based on a 238 urinary peptide pattern CAD238 [7]. CAD238 also revealed a prognostic potential for CAD endpoints including non-fatal AMI [12]. In the current multi-cohort study, we explored the urinary proteome profiles of participants from Australian, European and North American prospective cohorts to identify an ACS-specific pattern of urinary peptides that will allow prediction of future ACS events.

## Methods

### Study population

This study drew cases, comprising individuals with an ACS within 5 years post urine sampling with available urinary proteomic data, and sex- and age-matched controls without ACS from four separate studies conducted in Australia, Europe and North America. In total 218 proteomic data sets (109 incident ACS cases and 109 controls without ACS during follow-up) originated from the Australian Diabetes, Obesity and Lifestyle (AusDiab) study which is the largest Australian longitudinal population-based study examining the natural history of diabetes, heart disease and kidney disease [13]. Fourteen proteome profile data sets (7 ACS cases and 7 controls without ACS during follow-up) originated from the Flemish Study on Environment, Genes and Health Outcomes (FLEMENGHO) which is a prospective population-based study examining the potential effects of specific genes on blood pressure conducted in Northern Belgium [8,9]. Eight proteome profile data sets (4 ACS cases and 4 controls without ACS during follow-up) originated from the Coronary Artery Calcification in Type 1 Diabetes Study (CACTI) which is a longitudinal cohort study investigating the determinants of atherosclerosis in people with and without type 1 diabetes [14]. Twelve proteome profile data sets (6 ACS cases and 6 controls without ACS during follow-up) originated from the Hypertensive Atherosclerotic Cardiovascular Disease (HACVD) sub-study population of the Anglo-Scandinavian Cardiac Outcomes Trial (ASCOT) study [12]. The study was conducted in accordance with the principles of the Declaration of Helsinki and written informed consent was obtained from all the participants. The study was approved by the local ethics committee at the Medical School Hannover, approval number 3184–2016.

All participants were asymptomatic of coronary artery disease at the time of enrolment when urine samples were collected. Cardiovascular outcomes were adjudicated up to 5 years post urine sampling (see assessment of outcome section). The study population was split randomly into 2 groups (biomarker discovery cohort and validation cohort) by a ⅔ to ⅓ ratio. The biomarker discovery cohort was used to identify urinary peptide biomarkers which might potentially discriminate cases (individuals with future ACS events during follow-up) from controls (individuals without ACS during follow-up). 84 cases with 84 age- (within 5 years) and sex-matched controls were randomly selected for this purpose. The urinary peptide biomarkers thus identified were then applied to the remainder of the cases and controls for validation (validation cohort) in a blinded manner. 42 cases and 42 controls were used for validation. Thirty-six (28.6%) out of the 126 participants with an ACS event during the observation period of 5 years (cases) had a previous history of angina pectoris and/or AMI. In the other 90 participants it was the first cardiac event without a past history of known coronary artery disease. Out of the 126 control individuals 9 (8.3%) had previous angina pectoris and/or AMI but had no ACS event during the observation period.

### Assessment of outcome

For this study four outcomes were considered, non-fatal ACS (N = 67), fatal ACS (N = 58), ACS without information on fatality (N = 1) and no ACS during a follow-up time up to 5 years after urine sampling (controls). Non-fatal ACS was defined as either non-fatal AMI or new onset or worsening angina pectoris requiring hospitalization with angiographically documented coronary atherosclerosis or transient electrocardiographic changes of the ST-segment or T-wave without evidence for myocardial necrosis. AMI was defined as having at least two of: (i) a typical clinical presentation, (ii) ECG changes and (iii) cardiac enzymes rises (including creatine kinase and troponin) compliant with World Health Organisation MONICA criteria for myocardial infarction. Fatal ACS was defined from death certificate coding, using International Classification of Diseases Version 10 (ICD-10) codes I20-I25.

### Sample preparation and Capillary Electrophoresis–Mass Spectrometry (CE-MS) analysis

Urine sampling followed established standard operating procedures. Samples were kept frozen at -80°C, which has been shown to preserve proteomic profiles [15]. Proteomic analysis of all urine samples was performed by Mosaiques Diagnostics using the same protocol for all cohorts investigated. For proteomic analysis, 0.7 mL aliquot of urine was thawed immediately before use and diluted with 0.7 mL of 2 M urea, 10 mM NH_4_OH containing 0.02% sodium dodecyl sulfate. To remove higher molecular mass proteins, such as albumin and immunoglobulin G, the sample was ultra-filtered using Centrisart ultracentrifugation filter devices (20 kDa MWCO; Sartorius, Goettingen, Germany) at 3000 rcf until 1.1 ml of filtrate was obtained. This filtrate was then applied onto a PD-10 desalting column (GE Healthcare, Uppsala, Sweden) equilibrated in 0.01% NH_4_OH in HPLC-grade in H_2_O (Roth, Germany) to decrease matrix effects by removing urea, electrolytes, salts, and to enrich polypeptides present. Finally, all samples were lyophilized, stored at 4°C, and suspended in HPLC-grade H_2_O shortly before CE-MS analyses, as described previously [16].

CE-MS analyses were performed using a P/ACE MDQ capillary electrophoresis system (Beckman Coulter, Fullerton, USA) on-line coupled to a microTOF MS (Bruker Daltonics, Bremen, Germany) as described previously [16,17]. The ESI sprayer (Agilent Technologies, Palo Alto, CA, USA) was grounded, and the ion spray interface potential was set between –4 and –4.5 kV. Data acquisition and MS acquisition methods were automatically controlled by the CE *via* contact-close-relays. Spectra were accumulated every 3 s, over a range of *m/z* 350 to 3000. Accuracy, precision, selectivity, sensitivity, reproducibility, and stability of the CE-MS measurements were demonstrated elsewhere [16].

### Proteomics data processing

Mass spectral peaks representing identical molecules at different charge states were deconvoluted into single masses using MosaiquesVisu software [18]. Only signals with z>1 observed in a minimum of 3 consecutive spectra with a signal-to-noise ratio of at least 4 were considered. Reference signals of 1770 urinary polypeptides were used for CE-time calibration by locally weighted regression. For normalization of analytical and urine dilution variances, signal intensities were normalized relative to 29 ‘‘housekeeping” peptides with small relative standard. For calibration, linear regression was performed [16,19]. Deviation of CE migration time was controlled to be below 0.35 minutes after calibration. The resulting peak list characterized each peptide by its molecular mass (Da) and normalized CE migration time (minutes). Normalized signal intensity was used as a measure for relative abundance. All detected peptides were deposited, matched, and annotated in a Microsoft SQL database allowing further statistical analysis [20]. For clustering, peptides in different samples were considered identical if mass deviation was <50 ppm. Due to analyte diffusion effects, CE peak widths increase with CE migration time. In the data clustering process, this effect was considered by linearly increasing cluster widths over the entire electropherogram (19–45 min) from 2 to 5%.

### Sequencing of polypeptides

Identified prognostic biomarkers for ACS events were *in silico* assigned to the previously sequenced peptides from the Human urinary proteome database, version 2.0. Peptides from the Human urinary proteome database were sequenced as described elsewhere [21,22].

### Biomarker discovery

Peptides (< 20 kDa) present in urine as a result of naturally occurring protein degradation were investigated as potential biomarkers. For this investigation, statistical analysis of selected urinary proteome profiles was performed using non-parametric Wilcoxon rank sum test. Up to 2042 distinct peptides were analyzed in individual proteome profiles. Only peptides that were present at a frequency of 70% or higher in either case or control group were considered as potential biomarkers. Thus, the identified peptide biomarkers were independent of the cohort and potential population-specific genetic variability. The false discovery rate adjustments of Benjamini-Hochberg [23] were employed to correct for multiple testing. A *P*-value less than 0.05 was considered to be statistically significant.

In each of the 4 study cohorts, participants with verified ACS during follow-up were randomly assigned either to biomarker discovery or validation by a ⅔ to ⅓ ratio. The participants selected for biomarker discovery were representative of the cases in each cohort and matched to controls by age (within 5 years range) and sex. A match for cardiovascular (CV) risk based on 10-year cardiovascular disease risk prediction scores generated by the primary lipid Framingham model [24] between cases and controls was intended but not always possible (Table 1).

**Table 1:** Demographics and clinical features of the discovery cohort.

	control (N = 84)	ACS (N = 84)
**Women (%)**	36	35
**Age, years**	64 ± 11	64 ± 12
**Systolic pressure (mm Hg)**	136 ± 19	147 ± 23
**Diastolic pressure (mm Hg)**	72 ± 12	79 ± 13
**BMI (kg/m**^**2**^**)**	26 ± 4	29 ± 5
**Current smokers (%)**	10	24*
**Total cholesterol (mmol/L)**	6.0 ± 1.1	5.9 ± 1.3
**HDL cholesterol (mmol/L)**	1.5 ± 0.4	1.2 ± 0.3
**eGFR (ml/min/1.73m**^**2**^**)**	72 ± 11	70 ± 15
**Diabetes mellitus (%)**	13	29*
**Hypertension (%)**	45	69*
**History of cardiac events (%)**^**#**^	7	23*
**Median time to event (years)**	N/A	2.3 ± 1.5

BMI, body mass index; HDL high-density lipoprotein cholesterol; diabetes mellitus type I and II; hypertension was defined as blood pressure of ≥140 mmHg systolic, or ≥90 mm Hg diastolic, or use on antihypertensive drugs; N/A, not available; some data (e.g. smoking status) are not available for all individuals)

^#^ angina pectoris and/or AMI

Differences between cases and controls have been assessed by Mann-Whitney rank sum test and marked with an * when *P* < 0.05.

### Support vector machine (SVM) modelling

The classifier established by SVM modelling allows the classification of samples in the high dimensional data space. ACS-specific peptide biomarkers were combined into a single summary multidimensional classifier using the SVM-based MosaCluster proprietary software, version 1.7.0 [25]. Classification is performed by determining the Euclidian distance (defined as the SVM classification score) of the vector to a maximal margin separating hyperplane. The SVM classifier uses the log_2_ transformed intensities of x features (peptides) as coordinates in an N-dimensional space (N = 75 for the ACSP75 biomarker pattern). It then builds an N-1 dimensional hyperplane that spans this space by performing a quadratic programming optimization of a Lagrangian using the training labels only while allowing for samples to lie on the wrong side of the plane. For such mistakes in classification the SVM introduces a cost parameter C. Because non-separable problems in low dimensions may be separable in higher dimensions the SVM uses the Kernel-trick to transform the samples to a higher dimensional space. MosaCluster uses the standard radial basis functions as kernel. These functions are just Gaussians with the parameter ɣ controlling their width. The optimal parameters C and ɣ are found via e.g. cross validation error estimation using a lattice build by different values of these two parameters. SVMs are generally implemented in most popular data mining software, particularly the kernlab cran contributed R package is a versatile tool for building SVM based-classifiers [26].

### Statistical methods and determination of predictive potential

For biomarker discovery, the reported unadjusted *P*-values were calculated using the univariate non-parametric Wilcoxon rank sum test. Statistical adjustment due to the existence of multiple test sets was performed by applying the Benjamini-Hochberg false discovery rate corrections [27]. By maximizing Youden’s index based on exact binomial calculations carried out in MedCalc version 12.7.3.0 (MedCalc Software, Mariakerke, Belgium, http://www.medcalc.be), we determined optimal thresholds for the ACS classifier to differentiate individuals with and without future ACS and calculated sensitivity and specificity given as mean along with their 95% confidence intervals in brackets. We assessed the predictive capacity (discrimination) of models using Harrell’s c-statistic. The c-statistic estimates the probability of concordance between predicted risk and the observed order of events from a randomly selected pair of participants while accounting for censored data. A score of 1.0 indicates perfect discrimination and 0.5 indicates poor discrimination. The c-statistic and 95% confidence intervals (CI) from each model were estimated using the somersd package, respectively, in STATA (version 12.1, (StataCorp, College Station, TX, USA), as described [28].

For demographic data, means were compared using ANOVA and proportions by Fisher’s exact test. Statistical significance was a 1-sided significance level of 0.05.

We used Cox regression to compute standardized hazard ratios. The response variable used was the “hazard” of an ACS occurring and baseline characteristics considered as covariates in Cox regression were sex, age, current smoking status, body mass index, diabetes mellitus, hypertension (office blood pressure of ≥140 mmHg systolic, or ≥90 mm Hg diastolic, use of antihypertensive drugs and/or history of elevated blood pressure), estimated glomerular filtration rate (eGFR), total cholesterol, HDL cholesterol, and history of cardiovascular disease (angina pectoris, myocardial infarction, stroke). We identified covariates to be retained in the analyses by a step-down procedure, removing the least significant covariate at each step until all *P*-values of covariates were less than 0.05. All Cox models complied with the proportional hazards assumption.

To further evaluate the added predictive potential of the established prognostic algorithms (ACSP75 and ACSCP), we used the net reclassification improvement (NRI) and the integrated discrimination improvement (IDI) method [29]. We calculated the c-statistics, the NRI, and IDI considering the risk categories <10% (low), 10–19% (intermediate), and ≥20% (high) for the 10-year cardiovascular disease risk prediction scores generated by the primary multi-marker lipid Framingham model (FCVRS) [24]. The formulas for the calculation were as follows:

(a) model 1: logit(y=ACS case/control) = α0 + α1 x [study centre] + α2 x [FCVRS]

(b) model 2: logit(y=ACS case/control) = α0 + α1 x [study centre] + α2 x [FCVRS] + α3 x [ACSP75 score]

(c) model 3: logit(y=ACS case/control) = α0 + α1 x [study centre] + α2 x [FCVRS] + α3 x [ACSCP score]

## Results

### Identification of biomarkers in the discovery data set

**Fig 1:**
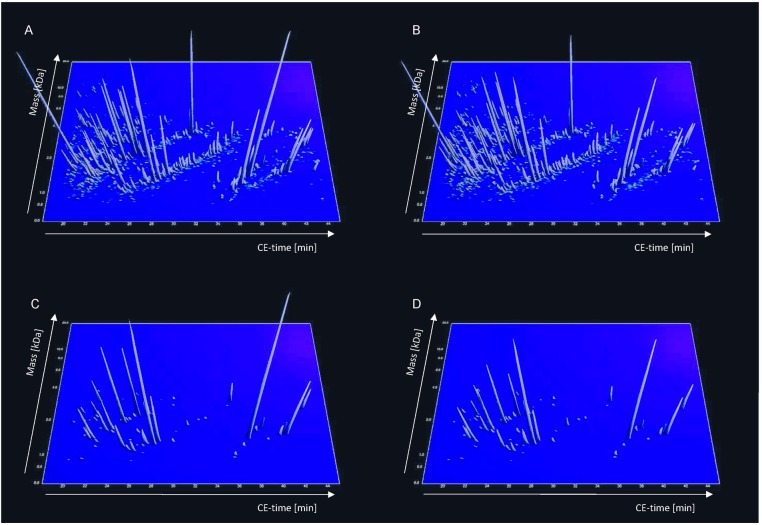
Urinary polypeptide patterns of control and individuals with ACS. Panels A and B: Compiled polypeptide patterns of the controls and individuals with ACS events during follow up after urine sampling examined in the training set; the molecular mass (0.6–20 kDa, on a logarithmic scale) is plotted against normalized migration time (18–45 min). Signal intensity is encoded by peak height and colour. Panels C and D: Distribution of potential biomarkers for ACS in controls and individuals with ACS events during follow up after urine sampling based on the ACS-specific peptide biomarker pattern. All statistically significant biomarkers are shown.

Proteomics data of all subjects involved in this study were listed in S1, S2, S3 and S4 Tables (supporting information). To identify ACS-specific prognostic urinary peptide biomarkers potentially discriminating between individuals with (cases) and without (controls) future ACS events, we compared the CE-MS-based urinary proteome profiles of 84 fatal and non-fatal ACS cases occurring within a mean time interval of 2.34 ± 1.48 years during follow-up after urine sampling and 84 age- and sex-matched controls. The clinical characteristics of these selected cases and controls are presented in Table 1. The previously published proteomic biomarker pattern characteristic for coronary artery disease CAD238 [7,12] discriminated between these patients and controls with a c-statistic of 0.574 (95% confidence interval) (0.515–0.633) with adjustment for time to event. Classification by the 10-year cardiovascular disease risk prediction scores generated by the composite multi-marker primary lipid Framingham model [24] resulted in a c-statistic of 0.636 (0.578–0.693) with adjustment for time to event. The central assessment of the current study is the univariate analysis (including correction for multiple testing) leading to the identification of 75 statistically significant (*P* < 0.05) peptide biomarkers enabling the discrimination between cases and controls (Fig 1), of which 54 (72%) were not part of the CAD238 biomarker pattern. 51 (68%) could be characterized by sequence and post-translational modifications (Table 2). The majority of the sequenced peptides originated from constituents of the extracellular matrix (ECM), i.e. fragments of various types of collagens, comprising type I (N = 43) and II (N = 3), respectively. Other identified peptides originated from apolipoprotein A-IV, complement C3, fibrillin-1, forkhead box protein O1, mucin-1, mucin-3, sarcalumenin or titin.

**Table 2:** Sequenced peptides constituting the ACS-specific peptide panel and their differential excretion between ACS and Controls.

Peptide ID	Protein name	Accession number	Sequence	CAD 238	DE^1^
31525	Apolipoprotein A-IV	P06727	FQDKLGEVNTY	y	
58084	Collagen alpha-1(I) chain	P02452	GPpGPpGKNGDDGEAGKpG		-1.35
90344	Collagen alpha-1(I) chain	P02452	GKNGDDGEAGKpGRpGERGPpGPQ		-1.04
23697	Collagen alpha-1(I) chain	P02452	DDGEAGKpGRpG		-1.07
82026	Collagen alpha-1(I) chain	P02452	GNSGEpGApGSKGDTGAKGEpGPVG	y	-1.14
70635	Collagen alpha-1(I) chain	P02452	NSGEpGApGSKGDTGAKGEpGP		1.06
79626	Collagen alpha-1(I) chain	P02452	NSGEpGApGSKGDTGAkGEpGPVG	y	1.04
72896	Collagen alpha-1(I) chain	P02452	SGEpGApGSKGDTGAKGEpGPVG		1.12
5675	Collagen alpha-1(I) chain	P02452	DGKTGPpGPA		-1.27
28561	Collagen alpha-1(I) chain	P02452	SpGPDGKTGPpGPA	y	-1.38
14906	Collagen alpha-1(I) chain	P02452	DGRpGPpGPpG		-1.27
127351	Collagen alpha-1(I) chain	P02452	AAGEPGkAGERGVpGPpGAVGPAGKDGEAGAQGPPGP		1.00
49332	Collagen alpha-1(I) chain	P02452	GPpGEAGKpGEQGVpGD		1.17
74902	Collagen alpha-1(I) chain	P02452	GPpGEAGkPGEQGVPGDLGApGP		-1.03
17694	Collagen alpha-1(I) chain	P02452	ApGDRGEpGpP		-1.12
32171	Collagen alpha-1(I) chain	P02452	ApGDRGEpGPpGPA	y	-1.05
81758	Collagen alpha-1(I) chain	P02452	ADGQpGAKGEpGDAGAKGDAGPpGP		1.00
85761	Collagen alpha-1(I) chain	P02452	ADGQpGAKGEpGDAGAKGDAGPpGPA		-1.06
77763	Collagen alpha-1(I) chain	P02452	DGQpGAKGEpGDAGAKGDAGPPGp	y	1.10
118224	Collagen alpha-1(I) chain	P02452	ESGREGApGAEGSpGRDGSpGAKGDRGETGPA		-1.06
78073	Collagen alpha-1(I) chain	P02452	AEGSpGRDGSpGAKGDRGETGPA		-1.40
43442	Collagen alpha-1(I) chain	P02452	VGPpGPpGPpGPPGPPS		1.03
44618	Collagen alpha-1(I) chain	P02452	VGPpGPpGPpGpPGPPS		1.01
34766	Collagen alpha-1(I) chain	P02452	PpGPpGPpGpPGPPS		-1.02
26113	Collagen alpha-2(I) chain	P08123	GppGPDGNKGEpG		1.08
41431	Collagen alpha-1(II) chain	P02458	GPpGKpGDDGEAGKPG	y	1.02
27517	Collagen alpha-1(II) chain	P02458	ApGEDGRpGPpGP		1.01
24502	Collagen alpha-1(II) chain	P02458	GpVGpAGGpGFpGA		
33973	Collagen alpha-1(II) chain	P02458	PVGpSGKDGANGIpG		-1.13
69769	Collagen alpha-1(III) chain	P02461	DGESGRPGRpGERGLpGPpG		1.04
117770	Collagen alpha-1(III) chain	P02461	GESGKPGANGLSGERGPPGpqGLpGLAGTAGEP		
121716	Collagen alpha-1(III) chain	P02461	GQPGVMGFpGPKGNDGAPGKNGERGGpGGpGpQ		
70413	Collagen alpha-1(III) chain	P02461	DGESGRpGRpGERGLpGPpG		1.00
61945	Collagen alpha-1(III) chain	P02461	GLpGTGGPpGENGKpGEPGp		1.22
70911	Collagen alpha-1(III) chain	P02461	GLpGTGGPpGENGKpGEPGpKG		1.12
18943	Collagen alpha-1(III) chain	P02461	SpGERGETGPp		-1.10
28747	Collagen alpha-1(III) chain	P02461	SpGERGETGPpGP		1.25
84542	Collagen alpha-1(III) chain	P02461	QNGEpGGKGERGAPGEKGEGGppG		1.01
71171	Collagen alpha-1(III) chain	P02461	GEPGGkGERGApGEKGEGGpPG		1.24
141804	Collagen alpha-1(V) chain	P20908	GEAGEPGLpGEGGpPGPKGERGEKGESGPSGAAGppGPKGP		-1.02
56053	Collagen alpha-2(V) chain	P05997	pGEGGKPGDqGVPGDPGAV	y	
102725	Collagen alpha-2(XI) chain	P13942	GNEGpSGPPGpAGSPGERGAAGSGGPIGpPG		1.00
132834	Collagen alpha-1(XVI) chain	Q07092	AGERGHPGAPGpSGSpGLPGVPGSMGDMVNYDEIK		1.29
42378	Collagen alpha-1(XVII) chain	Q9UMD9	AmGpPGPPGAPGPAGPAG		
99021	Collagen alpha-1(XXI) chain	F5GZK2	KGDPGLPGNpGYpGqPGQDGKPGYQG	y	
40091	Collagen alpha-1(XXII) chain	Q8NFW1	GpTGpPGKDGPnGPpG	y	
36156	Collagen alpha-1(XXV) chain	Q9BXS0	KGDqGqAGPPGppGP	y	
108021	Complement C3	P01024	EGVQKEDIPPADLSDQVPDTESETR		1.23
52189	Fibrillin-1	H0YND0	ECVDTDECSVGNPCGN	y	-1.06
37056	Forkhead box protein O1	Q12778	SGQEGAGDSPGSQFS	y	
7094	Hemoglobin subunit beta	P68871	SAVTALWGK		1.14
67263	Keratin; type II cytoskeletal 1	P04264	GSGGSSYGSGGGSYGSGGGGGGGRG		-1.41
8342	Mucin-1 subunit alpha	P15941	TTLASHSTK		-1.09
45445	Mucin-3A	Q02505	TSFSTIIWSSTPTI	y	1.19
71312	Protocadherin-12	Q9NPG4	FAERNPVEELTVDSPPVQ		1.23
123750	Rhox homeobox family member 1	Q8NHV9	EGGVNHENGmNRDGGmIPEGGGGNQEPRQQ		1.16
69979	Sarcalumenin	Q86TD4	EETEDANEEAPLRDRSH		-1.19
31480	Titin	Q8WZ42	KEADRGDSGTYD		1.08
65746	Uromodulin	P07911	SGSVIDQSRVLNLGPITR		-1.26
54438	Uromodulin	P07911	VIDQSRVLNLGPITR		1.05
48176	Uromodulin	P07911	IDQSRVLNLGPITR		-1.07

Only peptides discriminatory for ACS and characterized by sequence are shown (N = 61). The differential excretion (DE) of peptides between ACS and controls for the prognostic biomarker pattern ACSP75 has been calculated as follows: For mean MS amplitude (ACS) > mean MS amplitude (control): (mean ampl. (ACS) x frequency) / (mean ampl. (control) x frequency); For mean MS amplitude (ACS) < mean MS amplitude (control):—(mean ampl. (control) x frequency) / (mean ampl. (ACS) x frequency). For calculating means, values from all samples were used, considering 0 for undetected values; Peptide ID, peptide identifier annotated by the SQL database; CAD238, also present in the biomarker pattern CAD238; *P* in peptide sequences, oxidized prolines; m in peptide sequences, oxidized methionines.

### SVM modelling

The pattern of 75 ACS-specific peptide biomarkers was then applied to the urinary proteomic profiles used for biomarker discovery in subsequent support vector machine (SVM) based modelling of a proteomic prognostic ACS classifier. The resulting biomarker pattern **A**cute **C**oronary **S**yndrome **P**redictor 75 (ACSP75; radial basis function kernel with parameters C = 1638.4 and ɣ = 0.000256) allowed the classification of the ACS cases and controls of the discovery cohort with a sensitivity (95% confidence interval) of 83.3% (73.6–90.6) and a specificity (95% confidence interval) of 96.4% (89.9–99.3).

### Validation of the prognostic biomarker pattern ACSP75

**Fig 2:**
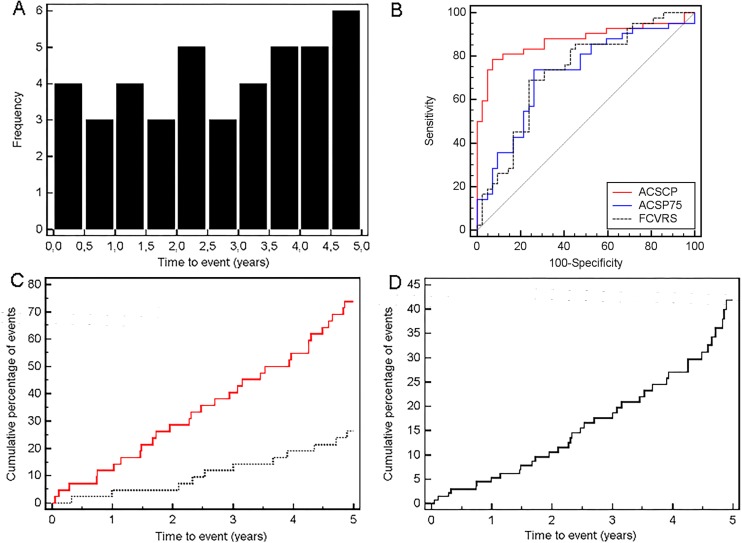
Performance of the ACSP75 urinary polypeptide classifier at follow up. A. Frequency histogram of ACS cases during follow-up. B. Receiver operating characteristic (ROC) curve for the validation set (N = 168). ROC analyses for prediction of ACS using the urinary ACS biomarker pattern ACSP75 (blue solid line), urinary composite classification by ACSCP (red solid line) and Framingham risk score (FCVRS; black spotted line) are shown. C. Kaplan Meier survival curve showing the cumulative percentage with an ACS event based on an ACSP75 score above (red solid line) and below (black spotted line) the threshold of 0.041. D. Multi-variable adjusted Cox proportional-hazards regression analysis of the same data sets based on ACSP75 score above and below the threshold of 0.041.

The clinical characteristics of the study participants in the validation data set, comprising 42 individuals with future ACS events (cases; mean time-to-event 2.74 ± 1.51 years; maximum time-to-event 4.89 years; Fig 2A) and 42 controls are presented in Table 3.

**Table 3:** Demographics and clinical features of the validation cohort.

	control (N = 42)	ACS (N = 42)
**Women (%)**	31	31
**Age, years**	68 ± 13	77 ± 9*
**Systolic pressure (mm Hg)**	147 ± 31	156 ± 22
**Diastolic pressure (mm Hg)**	78 ± 11	78 ± 13
**BMI (kg/m**^**2**^**)**	28 ± 4	27 ± 4
**Current smokers (%)**	2	7
**Total cholesterol (mmol/L)**	5.9 ± 1.6	5.9 ± 1.0
**HDL cholesterol (mmol/L)**	1.4 ± 0.3	1.4 ± 0.4
**eGFR (ml/min/1.73m**^**2**^**)**	72 ± 13	61 ± 17*
**Diabetes mellitus (%)**	19	43*
**Hypertension (%)**	69	95*
**History of cardiac events (%)** ^**#**^	7	38*
**Median time to event (years)**	N/A	2.7 ± 1.5

BMI, body mass index; HDL high-density lipoprotein cholesterol; diabetes mellitus includes type I and II; hypertension was an office blood pressure of ≥140 mmHg systolic, or ≥90 mm Hg diastolic, or use of antihypertensive drugs; eGFR, estimated glomerular filtration rate (MDRD formula); N/A, not available

^#^ angina pectoris and/or AMI

Differences between cases and controls have been assessed by Mann-Whitney rank sum test and marked with an * when *P* < 0.05; some data (e.g. smoking status) are not available for all individuals).

In comparison to controls, ACS cases were more likely to be older, hypertensive and have diabetes as well as a history of cardiovascular events (HCVE). Analysis of the ACS scores generated from the urinary proteome profiles by the ACSP75 classifier showed that ACSP75 discriminated between individuals with future ACS and controls with a sensitivity of 73.8% (58.0–86.1) and a specificity of 73.8% (58.0–86.1) based on an optimized threshold value of 0.041 for the ACSP75 scores. This discriminatory power was further demonstrated by a c-statistic of 0.664 (0.587–0.741) with adjustment for time to event. The positive likelihood ratio reached 2.82 (Fig 2B).

### Survival analysis for the biomarker pattern ACSP75

Longitudinal analysis of ACS events as outcomes in the validation cohort based on the prognostic ACSP75 scores as a dichotomous variable (0 = ACSP75 scores ≤ 0.041; 1 = ACSP75 scores > 0.041) by the Kaplan-Meier method revealed an unadjusted hazard ratio of 4.39 (2.14–9.01) for individuals with an ACSP75 score above the threshold of 0.041 (Fig 2C). In order to determine those clinical parameters relevant for ACS events as outcome/endpoint (response variable) in addition to the prognostic ACSP75 score as a dichotomous variable, and thus properly identify potential confounding factors, we performed stepwise Cox proportional hazards regression analysis (backwards, removal *P* at > 0.1). Based on clinical characteristics (Table 3), age, eGFR (estimated glomerular filtration rate), diabetes, hypertension and history of cardiovascular events were selected as variables approximating to the rule of 1 independent variable per 10 outcomes. The analysis revealed a variable-adjusted hazard ratio of 2.74 (1.32–5.70; *P* = 0.0072) for ACSP75 (Fig 2D) and a c-statistic of 0.743 (0.660–0.827) with adjustment for time to event. Finally, age and eGFR were identified as the only significant (*P* < 0.05) clinical parameters showing a hazard ratio of 1.08 (1.03–1.13; *P* = 0.0017) and 0.97 (0.95–0.99; *P* = 0.0098), respectively.

### Comparison of biomarker pattern ACSP75 to the Framingham risk score

In comparison to urinary proteomic prognostic classification, a prognostic classification of future ACS cases and controls in this study based on the well-recognized FCVRS model [24] resulted in a c-statistic of 0.644 (0.547–0.741) (Fig 2B) with adjustment for time to event. Thereby the common 20% high risk threshold for the Framingham score shows a sensitivity of 92.9% (80.5–98.5) but a specificity of only 31.0% (17.6–47.1). However, at the observed optimized risk threshold of 41.5%, the sensitivity was 69.5% (52.9–82.4) and the specificity was 76.2% (60.5–87.9). A comparison of prognostic discriminatory power of FCVRS with ACSP75 showed no significant difference in c-statistic. Adding ACSP75 scores to FCVRS did not provide any added prognostic value (Table 4; model 2 vs. 1) based on assessed incremental improvement of integrated discrimination improvement (IDI) or net reclassification improvement (NRI). Detailed formulas for the calculations for model 1 and 2 are provided in “Statistical methods and determination of predictive potential” section earlier.

**Table 4:** IDI and NRI for the prediction of ACS events by adding either ACSP75 or ACSCP scores to a basal model based on Framingham 10-year cardiovascular disease risk prediction scores (FCVRS).

	Model 2 vs. 1	Model 3 vs. 1
IDI	0.028 ± 0.015	0.284 ± 0.048
*P*-value IDI	*P* = 0.0645	*P* < 0.0001
NRI	0.024 ± 0.024	0.405 ± 0.119
*P*-value NRI	*P* = 0.3173	*P* = 0.0007

Model 2 vs. 1, improvement of basal FCVRS model (model 1) by adding ACSP75 scores; model 3 vs. 1, improvement of model 1 by adding ACSCP scores.

### Prognostic ACS classification by a composite score based on the biomarker pattern ACSP75

To test if combining the ACSP75 scores with clinical patient parameters and other CAD-related proteomic classifier scores further improves ACS prediction, we established the ACSP75-based composite prognostic **A**cute **C**oronary **S**yndrome **C**omposite **P**redictor (ACSCP). It combined the ACSP75 biomarker pattern scores with age (see Cox analysis above) as well as the CAD238 biomarker pattern scores specific for CAD [7,12] as parameters significantly (*P* < 0.05) contributing to ACS prediction based on logistic regression analysis. Other parameters like the eGFR showed no significant contribution. The formula for the calculation of the prognostic ACSCP classification score based on logistic regression analysis was as follows: ACSCP score = 0.2 x [ACSP75 score] + 2.6 x [CAD238 score] + 0.15 x [age]. When classifying the study participants of the validation data set with ACSCP, it showed a c-statistic of 0.751 (0.675–0.829) with adjustment for time to event with a sensitivity of 78.6% (63.2–89.7) and a specificity of 92.9% (80.5–98.5) based on a threshold of 10.256. The c-statistic of ACSCP was significantly higher than the one for the Framingham model (*P* = 0.021) showing a clear advantage in prognostic discriminatory power for ACSCP (Fig 2B). To further evaluate an added prognostic discriminatory power for ACSCP, we again assessed IDI and NRI (Table 4; model 3 vs. 1) and observed a significant incremental improvement of IDI and NRI compared to Framingham scoring. ACSCP therefore showed a positive likelihood ratio of 11.1. Detailed formulas for the calculations for model 1 and 3 are provided in “Statistical methods and determination of predictive potential” section earlier.

### Survival analysis for ACSCP

Longitudinal analysis of ACS events as outcomes in the validation cohort based on the prognostic ACSCP scores as a dichotomous variable (0 = ACSCP score < 10.256; 1 = ACSCP score > 10.256) by stepwise Cox proportional hazards regression analysis (backwards, removal *P* at < 0.1) again adjusted for age, eGFR, diabetes, hypertension and history of cardiovascular events revealed a variable-adjusted hazard ratio of 6.56 (2.36–18.25; *P* = 0.0003). None of the clinical parameters showed a significant (*P* < 0.05) contribution.

## Discussion

Peptides and some intact proteins circulating in the blood stream are excreted in urine through variable filtration in the kidney. Since this had previously led to the identification of urinary peptide biomarkers characteristic of atherosclerosis [30], particularly CAD [7,31,32], we hypothesized that urinary proteome/peptidome profiles contain peptide biomarkers indicative of different pathophysiological aspects in the progression of atherosclerotic plaques towards the inflamed, unstable, “vulnerable”, thin-cap fibroatheromas that are prone to rupture, and ultimately cause thrombotic occlusion of coronary arteries presenting as ACS. These peptides might originate either from plaques themselves or from activated circulating cells such as monocytes and platelets [33].

While prediction of ACS events in individuals of the discovery cohort using the already established biomarker pattern CAD238 was ineffective, state-of-the-art CE-MS analyses of their urinary proteome profile data allowed for the identification of ACS-specific urinary peptide biomarkers and the establishment a new prognostic classifier based on these biomarkers. This proteomic biomarker pattern ACSP75 proved to be capable of predicting the onset of an ACS up to 4.89 years before the event with a sensitivity of 73.8% in individuals who were asymptomatic at baseline. However, prediction of ACS with ACSCP surpassed prediction with ACSP75 suggesting that optimal prediction of ACS events can be achieved by integrating a urine peptide pattern characteristic for the presence of CAD and the age of an individual [7,12].

The peptide biomarkers characterized so far by their amino acid sequence predominantly originated from collagens, i.e. type I and III. Potential release of collagens via metalloproteinase activity, which is known to be upregulated in unstable and inflamed plaques, has been shown in human carotid endarterectomy specimens [34]. While surrounding endothelial cells in the tunica intima, collagens also contribute to the composition of the three dimensional network of vascular smooth muscle cells (VSMC), fibronectin, and proteoglycan-rich layers of the tunica media as well as the composition of the fibroblast-rich tunica adventitia [35,36]. Type I collagen can comprise approximately 60% of the total protein content of an atherosclerotic plaque and plays, in addition to proteoglycans, an active role in lipid retention [37]. Both, type I and III collagen are part of the complex and dynamic ECM of blood vessel walls thereby also contributing to the strength and integrity of the fibrous cap of a plaque as well as the modulation of cellular responses within it [35–37]. Moreover, a cap rich in fibrillar collagens and elastin confers stability to the whole plaque. Initial accumulation of ECM, particularly collagens, is further part of the fibrotic remodelling associated with hypertension and atherosclerosis [38,39]. The observed decrease of collagen type I fragments and increase of type III fragments in the excreted urine of individuals with a future ACS may therefore mirror atherogenic alterations of the ECM that contribute to plaque destabilization along with a weakening of the fibrous cap [37,40]. These processes may include altered collagen synthesis by endothelial cells in the intima and/or fibroblast and myofibroblast cells in the adventitia layer of blood vessels and therefore altered fibrillogenesis. They may also include altered covalent cross linking of collagens by lysyl oxidases (LOX) and/or oxidative modification e.g. by reactive aldehydes originating from oxidized low-density-lipoprotein [41] within the ECM. Collagens influence the function and activity of cells in the arterial wall, i.e. VSMCs and macrophages [35,38]. The composition of the ECM further directly regulates activities of proteases secreted by macrophages and VSMCs, which is highly relevant in the pathophysiology of plaque rupture [42].

The urinary biomarkers in our study seemed to be different from the plasma protein makers identified by Yin et al. A pattern of seven plasma proteins was found to be predictive of AMI by Yin et al, which included cyclophilin A, cluster of differentiation 5 molecule antigen-like cell-surface glycoprotein, mucin cell surface associated protein 18, collagen-α 1 [XVIII] chain, salivary α-amylase 1, C-reactive protein (CRP) and multimerin-2 [5]. The reason for this difference is not entirely clear although the urine proteome is expected to be inherently different from the plasma proteome due to several factors including variable metabolism and differential renal handling of some proteins and peptides. For example, CRP excretion in the urine is rare [43] and despite being an established serum marker of CAD, has not been seen in urine peptide patterns of CAD patients [7,31].

The previously described biomarker pattern CAD238 has been shown to be able to identify patients with stable coronary artery disease, as validated in patients undergoing elective surgical coronary revascularization [7]. Brown et al found CAD238 can also be useful in predicting the development of coronary artery disease in the future [12]. As the study cohort includes patients with a broad spectrum of CAD (patients with fatal CAD, non-fatal MI as well as patients just undergoing revascularization), it is not clear whether its prediction is specific for ACS or applies more broadly to coronary artery disease. Brown et al also found only some of the markers in the CAD238 panel were different between cases and controls, and hypothesized that these markers reflect earlier stages of CAD that had the potential to progress. It is interesting to see there is some overlap of the urinary biomarkers identified in ACSP75 of our study and the previously described CAD238 (Table 2). Some peptides derived from collagen alpha-1, fibrillin-1, mucin-3A were found in both CAD238 and ACSP75. It is possible that CAD238 predominantly identifies the patients who have or might develop CAD. However when atherosclerotic plaques become relatively unstable, the urinary peptide pattern somewhat changes to reflect the greater collagen breakdown in the fibrous cap, leaning towards ACSP75 pattern as discussed earlier. This would explain some overlap between the two panels. ACSP75 alone does not seem to perform better than the clinical Framingham score in predicting future cardiovascular events, highlighting that clinical parameters are still very important. But combining these two patterns (CAD238 for the presence of atherosclerotic plaques and ACSP75 for potential plaque instability) with age, which is the most important clinical risk factor in ACSCP, significantly increases the predictive value for future ACS events.

Several of the peptides identified as biomarkers for ACS fit well in the mechanistic concept of increased plaque instability in individuals with an increased risk of an ACS event. An association with the pathogenesis of atherosclerosis has been shown for circulating complement C3 [44], and with acute myocardial infarction for titin [45] and fibrillin-1 [46]. Notably, some of these proteins have also been identified in the urine of atherosclerotic mice [30], pointing out the possibility that a mouse model for unstable plaques [47] can be used to further investigate urinary biomarkers of plaque instability.

Our study adheres to the relevant guidelines of proteomic testing as the biomarkers described have a clear context of use which is “prediction of ACS” and performance of the biomarkers was not only evaluated in comparison to the current state of the art, but was also validated in a separate cohort in a blinded fashion. The main limitation of our study is the small sample size, especially in the validation cohort. While a positive discriminatory effect of ACSCP even in a small study sample serves as a “proof of concept”, it should be validated in a much larger independent cohort.

## Conclusion

A newly established urinary biomarker pattern reflects molecular pathological alterations associated with atherosclerotic plaque evolution towards “vulnerable” plaques, plaque rupture and ultimately thrombotic artery occlusion. This biomarker pattern potentially allows for a successful identification of individuals, who are at high risk of experiencing a future ACS event, thereby enabling timely preventative interventions. Further prospective studies exploring larger cohorts e.g. in the context of larger pharmacological trials are warranted to establish a highly attractive non-invasive concept of identifying individuals at risk, with the potential of initiating preventative measures and ultimately reducing cardiovascular mortality and morbidity.

## Supporting information

S1 Table Cohort classification for biomarker discovery and validation. A total of 252 individuals were used in this study. 0 represents individuals with no Acute Coronary Syndrome (ACS) used as controls and 1 represents individuals with ACS used as cases. For the biomarker discovery, the discovery cohort was used and the validation cohort was used for the ACS classifier validation.
(XLS)

S2 Table Mass spectrometry amplitudes of individuals. The amplitudes were given for all 252 individuals and all peptides (5605) identified by CE-MS.
(XLSX)

S3 Table All identified peptides using CE-MS. For each peptide, mean CE-migration time (min), and mass (Da) is given.
(XLS)

S4 Table (XLS)
